# Crosstalk of ferroptosis regulators and tumor immunity in pancreatic adenocarcinoma: novel perspective to mRNA vaccines and personalized immunotherapy

**DOI:** 10.1007/s10495-023-01868-8

**Published:** 2023-06-27

**Authors:** Yanlong Shi, Yizhu Wang, Hui Dong, Kaiyi Niu, Wenning Zhang, Kun Feng, Rui Yang, Yewei Zhang

**Affiliations:** 1 Hepatopancreatobiliary Center, The Second Affiliated Hospital of Nanjing Medical University, 210003 Nanjing, Jiangsu Province China; 2 School of Medicine, Southeast University, 210009 Nanjing, China

**Keywords:** Ferroptosis, FANCD2, Pancreatic adenocarcinoma, PD-L1, Immunotherapy, Prognosis, Drug sensitivity

## Abstract

Pancreatic adenocarcinoma (PAAD) is the eighth leading cause of cancer-related mortality that causes serious physical and mental burden to human. Reactive oxygen species accumulation and iron overload might enable ferroptosis-mediated cancer therapies. This study was to elusive novel ferroptosis regulator and its association with immune microenvironment and PD-L1 in PAAD. RNA-seq data and relevant information were obtained from The Cancer Genome Atlas and Genotype-Tissue Expression. The R packages “ggplot2” and “pheatmap” were used to the expression of 20 ferroptosis regulators between PAAD and normal tissues. The R package “ConsensusClusterPlus”, “survival”, “survminer”, “immunedeconv”, and TIDE algorithm performed consensus clustering, overall survival, progression-free survival, disease free survival, immune infiltration level, and immunotherapy responses between cluster 1 and cluster 2. The prognostic value was confirmed by the Kaplan–Meier curves, receiver operating characteristic curve, univariate and multivariate cox regression, and nomogram. Moreover, the relationship of FANCD2 and immunity, drug sensitivity was investigated by R package “ggstatsplot”, “immunedeconv”, “ggalluvial” and “pRRophetic”. Besides, the qRT-PCR, immunohistochemistry and western blotting detected the expression of FANCD2 in PAAD cell lines. Most ferroptosis regulators were up-regulated in PAAD, while the expression of LPCAT3, MT1G, and GLS2 was down-regulated in PAAD (P < 0.05), indicting there was a positively correlation among ferroptosis regulators. Based on clustering parameter, we identified cluster 1 and cluster 2, and cluster 2 had a better prognosis for patients with PAAD. The immune infiltration level of cluster 1 was higher in macrophage M1, myeloid dendritic cell, T cell CD4 + Th2, B cell, T cell CD8 + central memory, immune score, and microenvironment score than cluster 2 in PAAD. Moreover, FANCD2 was up-regulated in PAAD by public databases, immunohistochemistry, qRT-PCR and Western blotting, which had closely related to overall survival, immune microenvironment, and drug sensitivity. A novel crosstalk of ferroptosis exhibits a favourable prognostic performance and builds a robust theoretical foundation for mRNA vaccine and personalized immunotherapy. FANCD2 could be an effective for prognostic recognition, immune efficacy evaluation, and mRNA vaccine for patients with PAAD, providing a vital guidance for further study of regulating tumor immunity and vaccine development.

## Introduction

Pancreatic adenocarcinoma (PAAD), a devastating malignant disease that causes serious physical and mental burden to human, is the eighth leading cause of cancer-related mortality [1]. Pancreatic ductal adenocarcinoma (PDAC) is the most common pathological type of PAAD, accounting for more than 85% of pancreatic malignancies [2]. Numerous risk factors induced the occurrence and progression of PAAD including genetics, smoking, diabetes, chronic pancreatitis, and obesity [3]. Due to atypical earlier symptoms and rapid metastasis, most PAAD patients were diagnosed at advanced stages [4], so the postoperative 5-year survival rate of patients with PAAD is only about 10% [5]. Moreover, the majority of PAAD patients usually have a local or distant recurrence within 2 years after surgery [6]. Immunotherapy, as an emerging and promising treatment, has changed the prospective of therapeutic strategy in many solid tumors, such as melanoma and lung cancer [7]. Hence, it is urgent to comprehend the potential biological functions and molecular mechanisms in PAAD for developing novel drug targets.

Ferroptosis, first proposed in 2012, is a novel type of iron-dependent, non-apoptotic cell death and unlike apoptosis and necrosis [8]. When iron ions accumulated in cells under special conditions like oxidative stress, it can induce the production of reactive oxygen species (ROS) and lipid peroxidation to lethal levels, eventually lead to the rupture of cell membrane structure and death [9]. To support the specific biological behavior like increase proliferation, cancer cells have a higher demand for iron than normal tissues and it was confirmed that iron overload could contribute to ferroptosis in cancer. The high iron metabolism makes cancer cells more vulnerable to iron overload and ROS accumulation that might enable ferroptosis-mediated cancer therapies [10]. For instance, mutations of KRAS gene lead to a significant increase in intracellular ROS and then induced ferroptosis in PAAD [11]. Importantly, it has been confirmed that some drugs (artesunate and zalcitabine) can activate ferroptosis to suppress the proliferation of cancer cells in PAAD [12, 13]. Therefore, ferroptosis may be a vital factor to improve the efficacy of immunotherapy in PAAD. However, the underlying relationship between ferroptosis regulators and immune infiltration and PD-L1 remains elusive.

This study systematically explored the potential roles of ferroptosis regulators in multiple algorithms and programs based on molecular subtypes. Importantly, we first discovered and validated the expression of FANCD2, and comprehensively determined the association between different expression groups and prognosis, immune infiltration and immune therapy for patients with PAAD. FANCD2 could be a promising biomarker for PAAD patients, and its strong correlation with PD-L1 and tumor microenvironment might serve as an underlying condition for personalized treatment.

## Materials and methods

### Data acquisition

The RNA-seq data and relevant information were obtained from The Cancer Genome Atlas (TCGA) (https://portal.gdc.cancer.gov/) and Genotype-Tissue Expression (GTEx) (https://gtexportal.org/) databases, with 179 PAAD and 332 normal tissues. The key regulators of ferroptosis were retrieved from previous studies [14].

### Expression and correlation of ferroptosis regulators in PAAD

The R packages “ggplot2” and “pheatmap” were used to investigate the expression of 22 ferroptosis regulators between PAAD and normal tissues. Where different colors represented the expression and trend in different samples. In correlation analysis, the circles represent genes related to ferroptosis, the lines represent the interrelationships between genes, and the different colors of the circles represent different clustering categories. Among them, red and blue represent positive and negative correlation respectively.

### Screening of consensus clustering and evaluation of expression, prognosis and immunity

The R package “ConsensusClusterPlus” was conducted to consensus clustering analysis, and the optimal *k* value depended on repeating 100 samples from 80% of the population. The R package “pheatmap” presented the cluster heatmap of optimal *k* value. The R packages “survival” and “survminer” performed the overall survival, progression-free survival, and disease free survival between cluster 1 and cluster 2. To evaluate the immune capacity of two clusters, the R package “immunedeconv” was applied to investigate the level of immune infiltration cells [15]. Then, we extracted the expression values of immune checkpoint-related genes from RNA-seq data to observe the expression of immune checkpoint-related genes in different clusters. Moreover, The TIDE algorithm was used to predict potential immunotherapy responses in two clusters.

### Identification, validation, and development of key ferroptosis regulators

The Venn diagram was used to distinguish the core m6A regulator, and the mRNA expression of FANCD2 was invalidated by GEPIA, TCGA and GTEx databases, with 179 PAAD and 171 normal tissues, and 179 PAAD and 332 normal tissues, respectively. FANCD2 protein expression was analyzed by HPA database. Then, the module of “Pathological Stage Plot” was investigated the role in PAAD progression. The R package “maftools” was applied to download and visualize somatic genetic mutation of PAAD patients. The Kaplan–Meier survival curves performed the prognosis based on low- and high-expression groups, and the accuracy of prediction was assessed by Receiver Operating Characteristic Curve. The independent prognostic value of FANCD2 in PAAD patients was evaluated by univariate and multivariate Cox regression using R package “forestplot”. Moreover, the R package “rms” analyzed the survival possibility for 1- and 2-years based on the results of multivariate Cox regression.

### Analysis of FANCD2 with immune microenvironment and PD-L1 expression

The correlation between FANCD2 expression and immunity score, and tumor mutational burden were realized by R package “ggstatsplot”. Based on low- and high-expression groups, the relationship between FANCD2 expression and immune infiltrating cells was explored by R package “immunedeconv” using CIBERSORT algorithm, and the graphics were drawn using R package “ggplot2” and “pheatmap”. The correlation of FANCD2 expression and PD-L1 expression was assessed by R package “ggstatsplot”. Besides, the R package “ggalluvial” constructed a Sankey diagram for demonstrating the distribution trend of FANCD2 expression and clinical features, survival status. Each row represents a feature variable, different color represents different typing or stage, lines represent the distribution of the same sample in different feature variables [16]. The half-inhibitory concentration (IC50) is an important indicator to evaluate the efficacy of a drug or the response of a sample to treatment based on Genomics of Drug Sensitivity in Cancer (https://www.cancerrxgene.org/) [17]. The correlation between drugs and expression groups was analyzed by the R package “pRRophetic”, and the ridge regression estimated the half maximal IC50 of samples.

### Cell culture

The normal pancreatic cell (HPNE) and pancreatic cancer cell lines (CFPAC-1, SW1990, and BxPc-3) purchased from Procell Biotech (Wuhan, China). All cell lines were cultured with DMEM [10% fetal bovine serum (VivaCell, Shanghai, China)] in 5% CO_2_, 37 °C.

### Quantitative real-time polymerase chain reaction (qRT-PCR)

All cells RNA were extracted by the TRIzol reagent, and the reverse transcription performed by PrimeScript™ kit. The expression level of FANCD2 in different cells was detected by SYBR Green qPCR Mix. GAPDH was used as an internal control. The quantitative data were calculated as 2^−ΔΔCt^. The primer sequence was as follows:

### Immunohistochemistry

The immunohistochemistry performed FANCD2 protein expression in normal and PAAD tissues. We detected HNRNPC expression using “tissue” and “pathology” of modules in HPA database. All results were confirmed by two pathologists independently. Regents as following: FANCD2: Santa Cruz Biotechnology Cat#sc-20022, RRID:AB_2278211, dilution 1:10.

### Western blotting

Cells lysates and protease inhibitors lysed all cells, and protein concentrations were quantified by Bicinchoninic acid Protein Assay Kit. Proteins were detached by sodium dodecyl sulfatepolyacrylamide gel electrophoresis (SDS-PAGE) and transferred onto polyvinylidene fluoride (PVDF) membranes. The membranes were cultured with primary antibodies at 4 ℃ overnight, and then incubated with secondary antibodies (anti-rabbit, Proteintech, China) at indoor temperature. The membranes were visualized by chemiluminescence (Bio-Rad). The information of antibodies was included: FANCD2 (1:1000, Proteintech, China), GAPDH (1:3000, Proteintech, China).

### Statistical analysis

All the analysis methods and R packages were implemented by R version 4.1.0. The Wilcox test or Kruskal–Wallis test were used to explore the significance of two groups and three groups or more in PAAD and normal tissues, and two clusters. The Spearman correlation analysis performed the correlation between PD-L1 and ferroptosis regulators expression. P < 0.05 was considered statistically significant.

## Results

### Expression of ferroptosis regulators between PAAD and normal tissues

**Fig. 1:**
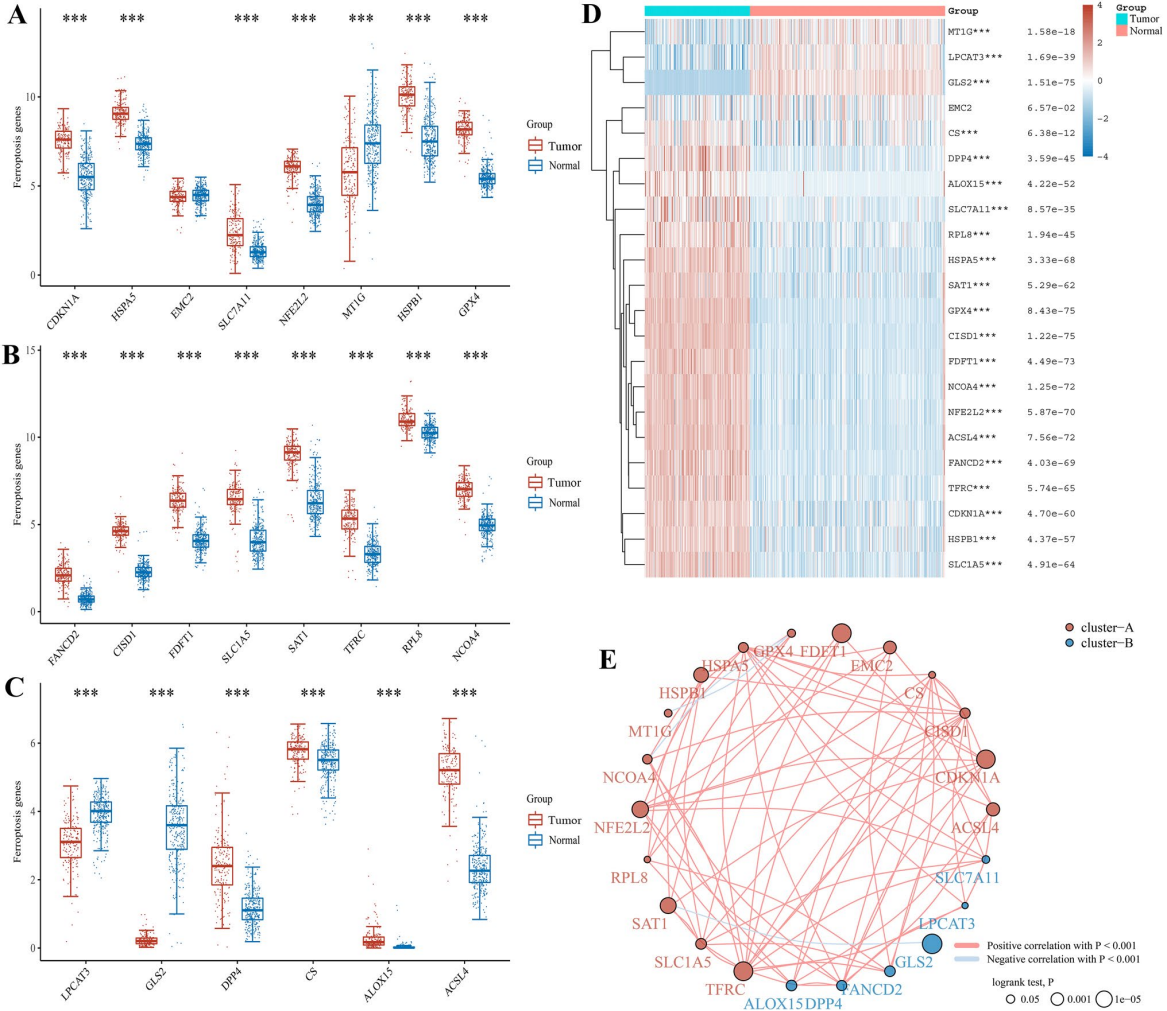
The expression and correlation of ferroptosis regulators in PAAD. **A-C** The expression of ferroptosis regulators in PAAD and normal tissues by Box plot analysis. **D** The expression of ferroptosis regulators between PAAD and normal tissues by Heatmap analysis. **E** The correlation combined with prognosis of ferroptosis regulators in PAAD by Spearman analysis. ^*^P < 0.05, ^**^P < 0.01, and ^***^P < 0.001

Based on 24 ferroptosis regulators, we analyzed their mRNA expression between 179 PAAD and 332 normal tissues in the TCGA and GTEx database. In Fig. 1A, most ferroptosis regulators were up-regulated in PAAD, including CDKN1A, HSPA5, SLC7A11, NFE2L2, HSPB1, GPX4, FANCD2, CISD1, FDFT1, SLC1A5, SAT1, TFRC, RPL8, NCOA4, DPP4, CS, ALOX15, and ACSL4 (*P* < 0.05), while the expression of LPCAT3, MT1G, and GLS2 was down-regulated in PAAD (*P* < 0.05). However, there was no differential expression of EMC2 between PAAD and normal tissues (*P* > 0.05). Then, the relevant expression heatmap was shown in Fig. 1B. Moreover, we indicated that there was a positively correlation in most ferroptosis regulators using correlation analysis (Fig. 1C). These findings demonstrated that ferroptosis regulators could serve as a key role in PAAD occurrence and progression.

### Screening and evaluation of optimal clusters and prognosis in PAAD

**Fig. 2:**
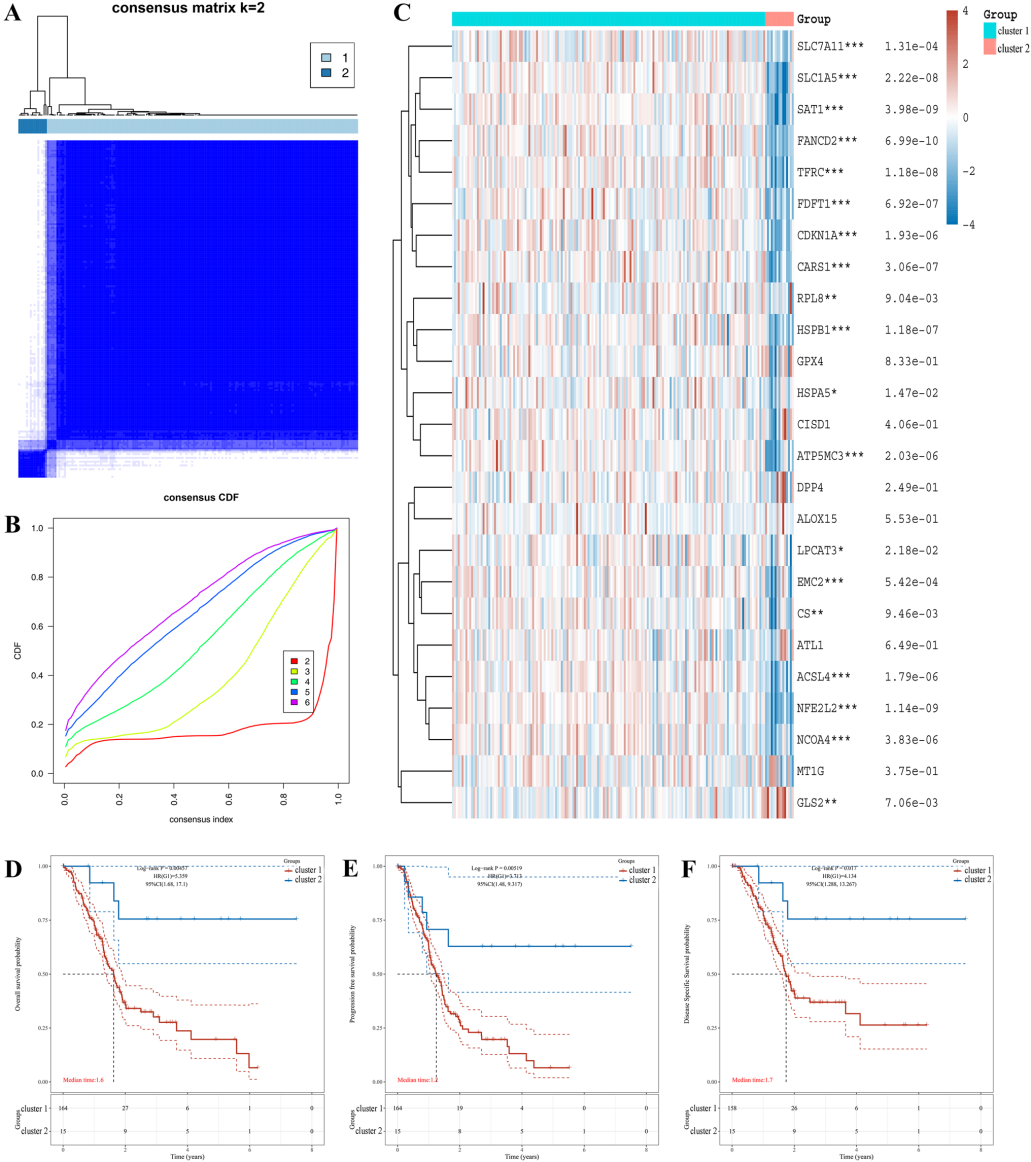
Consensus clustering analysis and prognosis of ferroptosis regulators in PAAD. **A** The optimal consensus clustering matrix k = 2.  **B** The cumulative distribution function (CDF) curves when cluster number varying from 2 to 6.  consensus clustering matrix k = 2. **C** The expression of ferroptosis regulators between cluster 1 and cluster 2 by heatmap analysis. **D**–**F** The overall survival (**D**), progression free survival (**E**), and disease-free survival (**F**) in two clusters for PAAD patients. ^*^P < 0.05, ^**^P < 0.01, and ^***^P < 0.001

To comprehend the value of ferroptosis regulators, we conducted to consensus clustering analysis for further study in PAAD. Based on cumulative distribution function curves and clustering heatmap, the optimal k value was selected as 2 for follow-up analysis (Fig. 2A, B). The baseline clinicopathological characteristics and relationship of two clusters were presented in Table 1. Moreover, the heatmap analysis suggested that the expression level of cluster 2 was lower than cluster 1 in PAAD (Fig. 2C). Besides, we investigated the prognosis of two clusters in PAAD, and the results suggested that patients with cluster 2 had a longer overall survival [*P* = 0.005, 95%CI (1.68, 17.1)] (Fig. 2D), progression-free survival [*P* = 0.005, 95%CI (1.48, 9.317)] (Fig. 2E), and disease free survival [*P* = 0.017, 95%CI (1.288, 13.267)] (Fig. 2F) than those in cluster 1. These results revealed that consensus clustering might preliminarily stratify the risk of PAAD patients.

**Table 1:** Clinical characteristics of two clusters of patients with PAAD

Characteristics	Cluster 1 (n = 164)	Cluster 2 (n = 15)	*P* value
Status			
Alive	74	12	0.020
Dead	90	3	
Age			
Mean (SD)	64.7 (11)	64.1 (10.2)	0.852
Gender			
Female	73	7	1.000
Male	91	8	
T stage			
T1	6	1	0.002
T2	18	6	
T3	137	6	
T4	2	1	
TX		1	
Not known	1		
N stage			
N0	46	4	0.007
N1	112	8	
N1b	4		
NX	2	2	
Not known		1	
M stage			
M0	75	5	0.449
M1	5		
MX	84	10	
pTNM			
IA	5		0.001
IB	10	5	
IIA	28		
IIB	112	7	
III	2	1	
IV	5		
I		1	
Not known	2	1	
Grade			
G1	22	9	0.000
G2	94	2	
G3	46	2	
G4	1	1	
GX	1	1	
Smoking			
No	58	8	0.524
Yes	73	6	
Not known	33	1	
Radiation			
No	92	11	0.690
Yes	30	2	
Not known	42	2	

### Depiction of the immune microenvironment and PD-L1 expression between two clusters

**Fig. 3:**
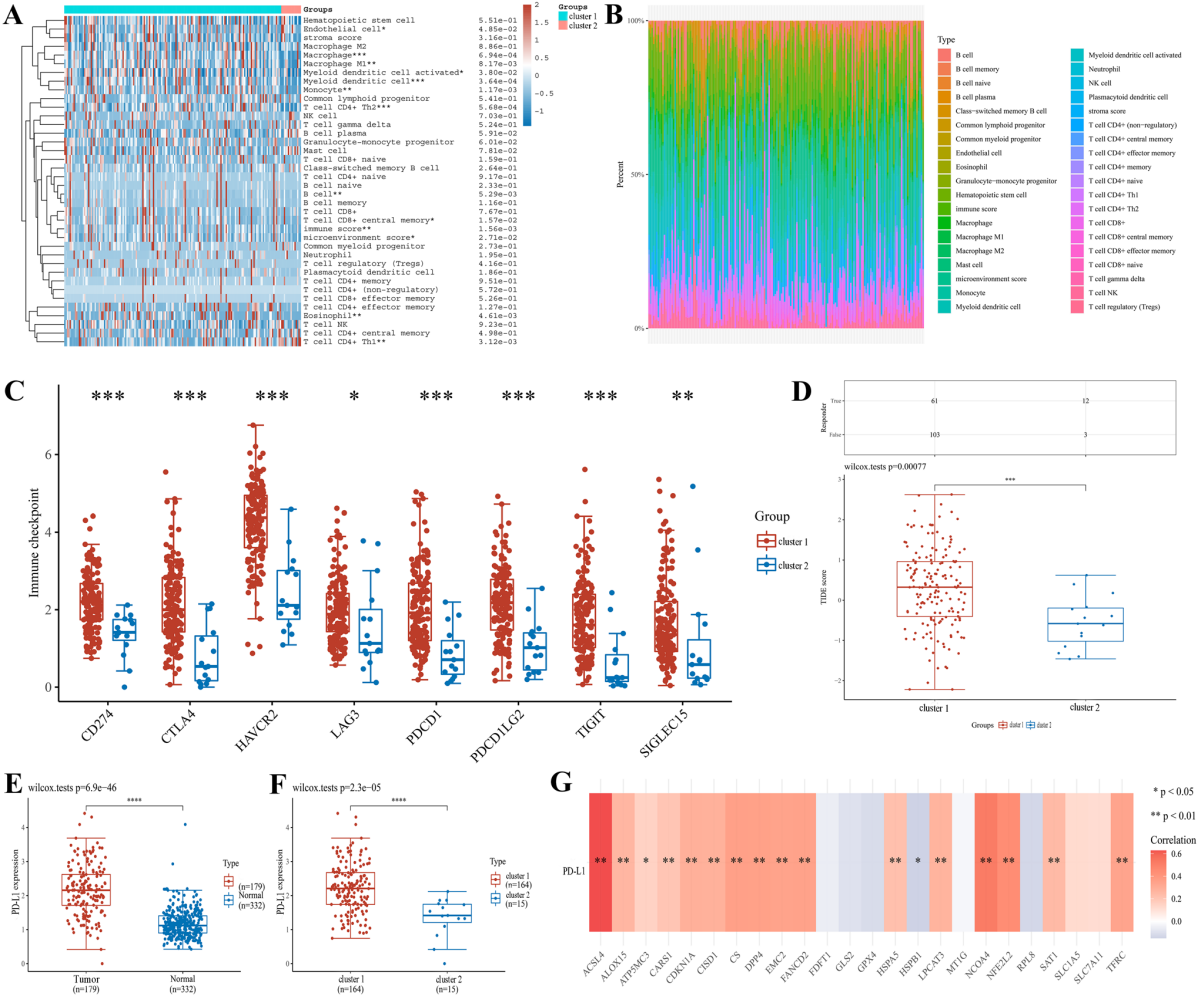
Relationship of cluster subtypes with immune infiltration level and PD-L1 in PAAD. **A** The expression level of immune infiltration cells in cluster 1 and cluster 2. **B** The proportion of immune infiltration cells in cluster 1 and cluster 2. **C** The association of immune checkpoint and cluster subtypes. **D** The expression of TIDE score in cluster 1 and cluster 2. **E** PD-L1 expression in PAAD and normal tissues. **F** PD-L1 expression in cluster 1 and cluster 2. **G** The correlation between PD-L1 expression and ferroptosis regulators in PAAD. ^*^P < 0.05, ^**^P < 0.01, and ^***^P < 0.001

Based on the above two clusters, we further investigated their relationship in immune infiltration cells, immune checkpoints, tumor mutational burden, and immunization checkpoint blockade. In Fig. 3A, The immune infiltration level of cluster 1 was higher in macrophage, macrophage M1, myeloid dendritic cell, T cell CD4 + Th2, B cell, T cell CD8 + central memory, T cell CD4 + Th1, eosinophil, immune score, and microenvironment score than cluster 2 in PAAD. Then, the proportion of each immune infiltrating cell was listed in Fig. 3B. The main eight immune checkpoints were include in this study, and the results suggested the expression of immune checkpoints was significantly different between cluster 1 and cluster 2 (Fig. 3C). Importantly, immune checkpoint blockade is gradually shifting treatment patterns in cancers [18]. The responsiveness of two clusters to immune checkpoint inhibitors was predicted by Tumor Immune Dysfunction and Exclusion (TIDE) algorithm. We found that cluster 1 had a high immune response score, reflecting PAAD patients treated with immune checkpoint blockade have poor prognosis (Fig. 3D). Moreover, we explored concretely the correlation between two clusters and PD-L1. PD-L1 expression was up-regulated in PAAD (Fig. 3E) and cluster 1 (Fig. 3F) compare to normal tissues and cluster 2, respectively. Besides, the correlation heatmap analysis indicated that most ferroptosis regulators were positively correlated with PD-L1, while HSPB1 was negatively correlated with PD-L1 in PAAD (Fig. 3G). These results implied that the two clusters might helpful to distinguish the response to immunotherapy in PAAD patients.

### Identification and validation of core ferroptosis regulator FANCD2 in PAAD tissues and cells

**Fig. 4:**
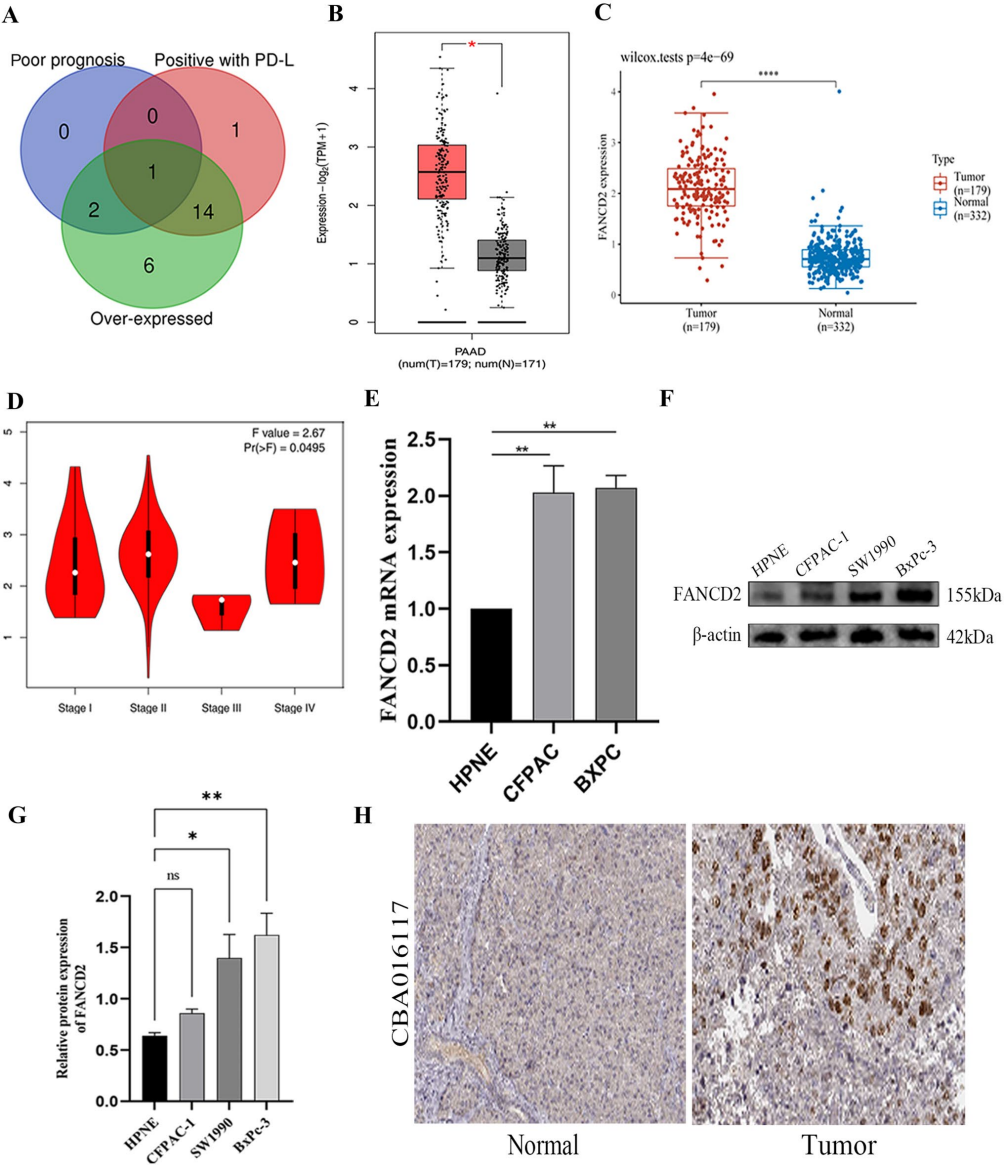
Identification and validation of core ferroptosis regulators FANCD2 in PAAD. **A** Identification of interaction gene FANCD2 by Venn graph. **B** The expression of FANCD2 in 179 PAAD and 332 normal tissues. **C** Up-regulated expression of FANCD2 in 179 PAAD and 171 normal tissues. **D** The relationship of FANCD2 expression and tumor stages. **E** FANCD2 mRNA expression was over-expressed in CFPAC-1 and BxPc-3 cell lines by qRT-PCR analysis. **F**–**G** FANCD2 protein expression was up-regulated in SW1990 and BxPc-3 cell lines by Western blotting. **H** FANCD2 was positively expression in PAAD compared to normal tissues by immunohistochemistry analysis. ^*^P < 0.05, ^**^P < 0.01, and ^***^P < 0.001

To better find core ferroptosis regulator in PAAD, an intersection analysis demonstrated that FANCD2 is the only interaction gene among genes with poor prognosis, positive with PD-L1, and over-expressed expression (Fig. 4A). Then, we invalidated FANCD2 expression in TCGA, GTEx, and GEPIA database, and the results suggested FANCD2 expression in PAAD was higher than normal tissues (*P* < 0.05) (Fig. 4B, C). In Fig. 4D, we also found FANCD2 expression was associated with pTNM stages. Moreover, the qRT-PCR analysis suggested that FANCD2 mRNA was up-regulated in CFPAC-1 and BxPc-3 cell lines than HPNE cell line (Fig. 4E). In Western blotting analysis, the protein expression of FANCD2 was increased in SW1990 and BxPc-3 cell lines than HPNE cell line (Fig. 4F, G). Besides, we investigated the protein expression of FANCD2 in PAAD. Compared with normal tissues, FANCD2 protein was a strong positive expression in nuclear in PAAD (Fig. 4H).

### Independent prognostic value of FANCD2 in PAAD

**Fig. 5:**
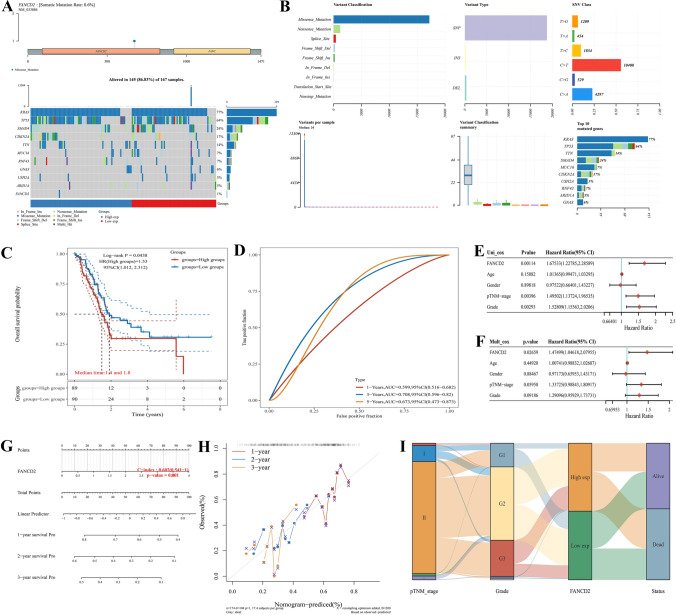
Mutant landscape and independent prognostic factors of FANCD2 in PAAD. **A** Oncoplot analysis of somatic landscape in HCC. **B** The variant classification, type and SNV class by cohort summary plot. **C** Kaplan–Meier survival curve presented the prognosis of FANCD2 in low- and high-expression groups. **D** The predictive ability of FANCD2 evidenced by the AUC value of ROC curve in 1-, 3-, and 5-years. **E** The univariate Cox regression between the relationship of FANCD2 expression and clinicopathological features. **F** The multivariate Cox regression between the association of FANCD2 expression and clinicopathological features. **G** Nomogram was constructed based on differences variables of multivariate Cox regression. **H** The prediction performance of nomogram in 1-, 2-, and 3-years by calibration curve. **I** The relationship of FANCD2 expression groups and stage, grade, and survival status in PAAD by Sankey diagram. ^*^P < 0.05, ^**^P < 0.01, and ^***^P < 0.001

To elucidate the independent prognostic value of FANCD2, we first investigated the genomic mutation of FANCD2 in PAAD. In Fig. 5A, FANCD2 had somatic mutation rate of 0.6%, and the mutation type was missense mutation. The main mutated genes in PAAD patients were KRAS, TP53, and SMAD4, with proportion of 77%, 64%, and 24%, respectively (Fig. 5B). Then, the cohort summary plot showed the variant distribution by variant classification, types and SNV class. Moreover, based on low- and high-expression groups, we found the low-expression group had a longer survival than high-expression group [P = 0.044, 95% CI (1.012, 2.312)] (Fig. 5C). The AUC values of ROC curves was 0.599, 0.708, 0.673 in 1-, 3-, and 5-years (Fig. 5D). The univariate and multivariate Cox regression analysis demonstrated FANCD2 was an independent prognostic factor in HCC (Fig. 5E, F). Accordingly, we constructed the nomogram to determine the survival probability in 1-, 2-, 3-years. The C-index is 0.602, suggesting might serve as an effective predictive application (Fig. 5G, H). Besides, the Sankey diagram verified the high-expression was associated with high stage, high grade, and poor prognosis in PAAD (Fig. 5I). These results showed that FANCD2 was an independent prognostic factor for PAAD patients.

### Correlation of FANCD2 expression and immune microenvironment, drug sensitivity

**Fig. 6:**
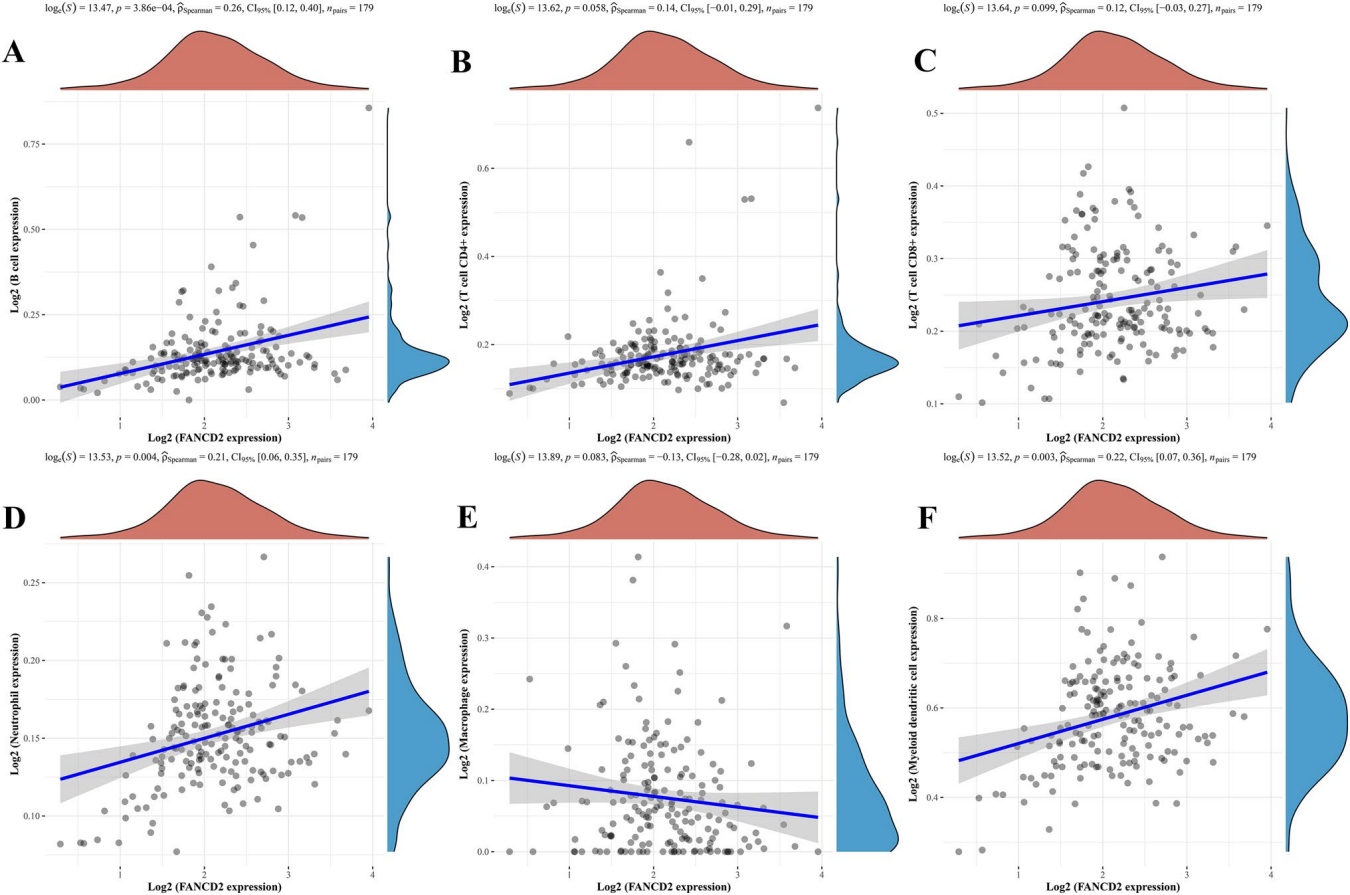
Correlation analysis between FANCD2 expression and immune cell infiltration in PAAD. **A**–**F** The correlation of FANCD2 expression and immune infiltration cells. ^*^P < 0.05, ^**^P < 0.01, and ^***^P < 0.001

**Fig. 7:**
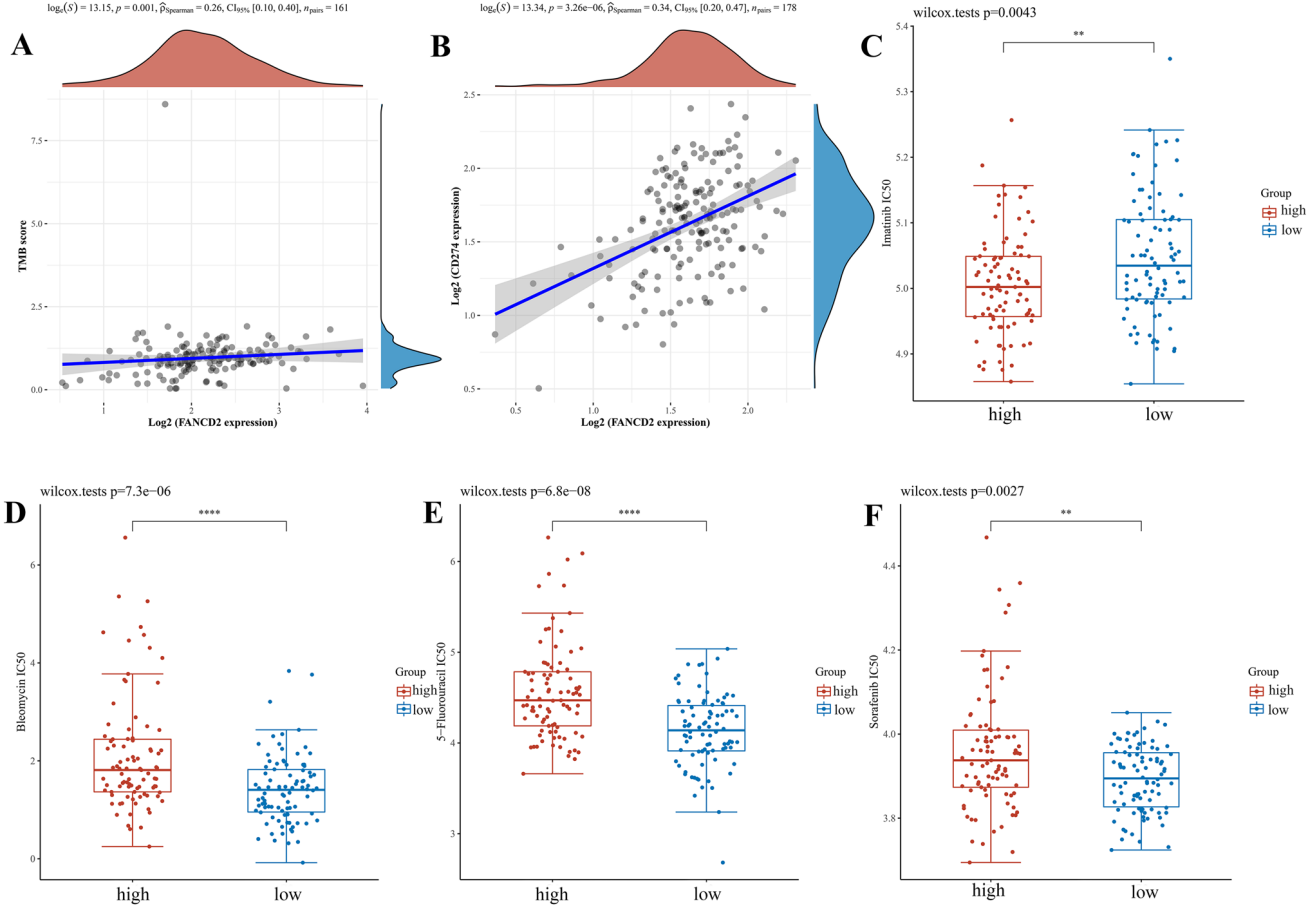
Correlation analysis between FANCD2 expression and FANCD2 expression and TMB score, PD-L1 expression, and drug sensitivity in PAAD. **A** Analysis of FANCD2 expression and TMB. **B** Correlation analysis of FANCD2 expression and PD-L1. **C**–**F** The association between FANCD2 expression and Imatinib, Bleomycin, 5-Fluorouracil, and Sorafenib. ^*^P < 0.05, ^**^P < 0.01, and ^***^P < 0.001

In Fig. 6A–F, correlation analysis indicated that FANCD2 expression was correlated with B cell, neutrophil, myeloid dendritic cell. Then, we investigated the relationship between FANCD2 expression and TMB score and PD-L1 expression, and the result exhibited a positive correlation (Fig. 7A, B). Furthermore, the relationship of drug sensitivity and FANCD2 expression groups was evaluated (Fig. 7C–F). There were significant differences in IC50 of drugs between low- and high-expression groups in PAAD patients, including Sorafenib, 5-Fluorouracil, and Bleomycin. Interesting, the sensitivity of PAAD patients in low expression group to Imatinib was higher than that in high expression group. Therefore, FANCD2 expression could play a critical role in immunotherapy response.

## Discussion

Since the concept of ferroptosis was first proposed by Dixon et al. [19], it has been found to participate in tremendous pathological processes, and increasing studies about the association between ferroptosis and cancers have been performed. As one of the most lethal malignancies worldwide, the interaction between ferroptosis and PAAD also attracts much attention. A study showed that the deletion of SLC7A11 can induced tumor-selective ferroptosis and inhibited PAAD progression [20]. Additionally, previous studies demonstrated that ferroptosis associates with tumor immunotherapy in pancreatic cancer. For example, Dihydroartemisinin (DHA) can induce PAAD cells ferroptosis through p53-AOLX12 pathway, and DHA treatment activated antitumor immunity in PAAD via inhibiting M2 cells and increasing CD8 T cells, NK cells and NKT cells [21]. Furthermore, Wang et al. found that CD8 + T cells can induce ferroptosis via releasing IFN-γ, which down-regulates the expression of Xc-complex [22]. Therefore, it is important to fully elusive novel ferroptosis regulator and its association with immune microenvironment and PD-L1 in PAAD.

In this study, we systematically investigated the expression of 24 ferroptosis regulators in PAAD and normal tissues by integrating multiple databases. Then, based on these profiles, two clusters (cluster 1 and cluster 2) were identified by consensus clustering, and was associated with clinicopathological characteristics, including status, T stage, N stage, pTNM, and grade. The Kaplan–Meier curves analysis demonstrated cluster 2 had a longer OS, PFS, and DFS, showing PAAD patients in cluster 2 was correlated with better prognosis. Moreover, a reasonable identification and validation first discovered that ferroptosis regulators FANCD2 was up-regulated in PAAD by public databases, immunohistochemistry, qRT-PCR and western blotting, which had closely related to overall survival, immune microenvironment, and drug sensitivity.

FANCD2, a class of Fanconi anemia protein, participates in endonucleolytic notch, HR pathway, and translation synthesis, thereby maintaining DNA damage repair and genome stability [23, 24]. Studies have demonstrated that FANCD2 has a strong resistance to tumor suppression by regulating DNA repair activity in hepatocellular carcinoma cells in vitro [25]. It is confirmed that elevated FANCD2 was an independent prognostic factor, and was associated with lymph node metastasis, tumor size and stage in tumors [26, 27]. Recently, FANCD2 has been reported to modulate ferroptosis negatively by regulating iron-dependent lipid peroxidation and iron metabolism, resulting a sensitive to ferroptosis induction treatment for tumors [8]. Increasing evidence indicated that FANCD2, a critical gene involved in autophagy-dependent ferroptosis, augmented the response of immunotherapy by activating P53 mutation in LUAD [28]. In our study, combined with ferroptosis regulators, we first discovered FANCD2 mRNA and protein expression was over-expressed in CFPAC-1 and BxPc-3 cell lines compared with HPNE cell. Moreover, FANCD2 could be an independent prognostic indicator in PAAD using univariate and multivariate analysis. Although FANCD2 might be a novel biomarker in the occurrence and progression of PAAD, the potential functions and mechanism should be further undertake.

The tumor microenvironment tend to affect the clinical outcome and prognosis of tumor patients. Immunotherapy is widely used in the treatment of various cancers, such as immune checkpoint and cyclin kinase inhibitors [29]. In comparison to cluster 2, we discovered that tumor immune cells, such as T cell CD4 + Th2, B cell, Macrophage M1, T cell CD8 + central memory, and T cell CD4 + Th1, had a higher level in cluster 1. A higher proportion of immune score and microenvironment score indicated cluster 1 was associated with multiple immune cells and immune-related pathways. Previous studies have demonstrated that CD8 + T cells could be build ferroptosis sensitization in cancer cells by secretion of IFNγ [22]. TIDE score is a promising biomarker to predict tumor immunotherapy response precisely [30]. Notably, there was an obvious difference in TIDE between cluster 1 and cluster 2, suggesting that patients in cluster 1 had a higher potential of tumor immune evasion and less likely to benefit from the therapy of anti-PD-1. Moreover, our results found that the expression level of PD-L1 is over-expressed in tumors and cluster 1, which further clarified the worse prognosis of PAAD patients with above groups.

The most interesting and significant finding was the association between FANCD2 and immune microenvironment, PD-L1, and drug sensitivity in PAAD. In this study, we identified FANCD2 expression had a strong correlation with B cell, Neutrophil, TMB, and PD-L1 in PAAD, which was closely related to tumor immunity. The ferroptosis-mediated ICD inducer combined with anti-PD-L1 was revealed to suppress tumor growth and regulate immune responses [31]. Increasing studies emerged the regulation between ferroptosis and drug sensitivity, resulting it can make cancer cells more sensitive to ferroptosis. Our study suggested that patients with high expression groups were more sensitive to Sorafenib, 5-Fluorouracil, and Bleomycin, which provide a promising therapeutic strategy in personalized treatment. To date, we first discovered the potential role of FANCD2 in immune and drug sensitivity, and future studies need to further clarify its relevance and mechanism.

There are still several limitations in this study. Firstly, owing to the heterogeneity of cancers, prospective, multi-center studies are still needed to further validate in PAAD patients to guarantee the stability and accuracy of molecular subtypes. Secondly, although we first discovered and invalidated the roles of ferroptosis regulators FANCD2 in PAAD for prognosis, immune infiltration and immune therapy, it is necessary to determine the potential biological functions of FANCD2. Thirdly, our study preliminarily identified a strong correlation between FANCD2 and PD-L1, but their mechanism in immunotherapy remains to be fully explored.

## Conclusion

Our study developed a novel molecular subtype in PAAD based on ferroptosis regulators which exhibited a favorable prognostic performance and built a robust theoretical foundation for mRNA vaccine and personalized immunotherapy. Moreover, we first discovered and validated FANCD2 was a promising biomarker and could be an effective for prognostic recognition, immune efficacy evaluation, and mRNA vaccine for patients with PAAD, providing a vital guidance for further study of the correlation between FANCD2 and PD-L1 on mRNA vaccine and personalized immunotherapy.

## Data Availability

All datasets and materials used in this study are included within the manuscript and available from the corresponding author on reasonable request.
